# Tetrahydrocannabinol Intoxication from Food at a Restaurant — Wisconsin, October 2024

**DOI:** 10.15585/mmwr.mm7427a2

**Published:** 2025-07-24

**Authors:** Amanda Kita-Yarbro, Stefanie Moccero, Katie Brobston, Jacob Goebel, Janice Block Banks, Christy Vogt, Casey Schumann, Katarina M. Grande, Julia Olsen, Bonnie Armstrong

**Affiliations:** 1 Public Health Madison & Dane County, Madison, Wisconsin.

**Summary**

**What is already known about this topic?**

Tetrahydrocannabinol (THC), a psychoactive substance found in *Cannabis sativa* plants, including varieties such as hemp, is increasingly being used in consumer products.

**What is added by this report?**

During October 22–24, 2024, at least 85 persons, ranging from age 1–91 years, ate food from a restaurant in Wisconsin and experienced symptoms consistent with THC intoxication. The restaurant was in a building with a cooperative (i.e., shared) kitchen used by a state-licensed vendor who produced edible THC products. The restaurant mistakenly used THC-infused oil from the cooperative kitchen to prepare dough.

**What are the implications for public health practice?**

Clinicians and public health practitioners should be alert to the possibility of mass THC intoxication events via food.

## Abstract

Tetrahydrocannabinol (THC), a psychoactive substance found in *Cannabis sativa* plants, including varieties such as hemp, is increasingly being used in consumer products. On October 24, 2024, local emergency medical services reported to Public Health Madison & Dane County (PHMDC) in Wisconsin that since October 22, they had transported seven persons to a local hospital for various symptoms, including dizziness, sleepiness, and anxiety. All seven persons reported having recently eaten food from the same local restaurant. Investigation by PHMDC determined that on October 22, the restaurant had run out of cooking oil and used oil from a cooperative (i.e., shared) kitchen located in the same building. One of the vendors who used the kitchen made edible products using hemp-derived Δ^9^-THC. On October 24, PHMDC posted a food and symptom history questionnaire on its website and shared the link via press release and social media. Among 107 responses that were considered valid, 85 persons met the following case definition of THC intoxication: 1) ate pizza, garlic bread, cheese bread, or a grinder (submarine sandwich) purchased from the restaurant during October 22–24 and 2) reported at least one symptom of THC intoxication that began within 5 hours after eating the restaurant’s food, defined as dizziness, sleepiness, anxiety, short term memory impact or time distortion, increased heart rate, nausea, paranoia, panic attack, increased blood pressure, vomiting, or hallucinations. Clinicians and public health practitioners should be alert to the possibility of mass THC intoxication events via food. Health care providers, public health professionals, and emergency responders should consider THC intoxication in persons with sudden onset of symptoms such as dizziness, sleepiness, anxiety, altered reality perception, increased heart rate, nausea, or other symptoms of THC ingestion. Regulations regarding practices such as standard, clear labeling and locked storage for ingredients containing THC, might decrease the risk for unintentional THC exposure at licensed food businesses.

## Investigation and Results

Tetrahydrocannabinol (THC) is a psychoactive substance found in *Cannabis sativa* plants, including varieties such as hemp; concentrations are lower in the hemp plant. On October 24, 2024, local emergency medical services reported to Public Health Madison & Dane County (PHMDC) in Wisconsin that since October 22, they had transported seven persons to a local hospital for various symptoms, including dizziness, sleepiness, and anxiety. All seven persons reported having recently eaten food from the same local restaurant. Emergency services conducted carbon monoxide testing in two residences of symptomatic persons and at the restaurant, and the test results were negative. The seven persons who were transported were treated in the emergency department (ED) of a Stoughton, Wisconsin hospital with symptoms of THC intoxication. Later that day, one of the ED patients, who had eaten pizza from the restaurant the previous day (October 23), contacted PHMDC at the suggestion of the hospital and reported receiving a positive THC test result without having knowingly consumed THC. PHMDC initiated an outbreak investigation. This investigation was determined by PHMDC to be nonresearch public health surveillance and therefore did not require institutional review board review.

**FIGURE 1:**
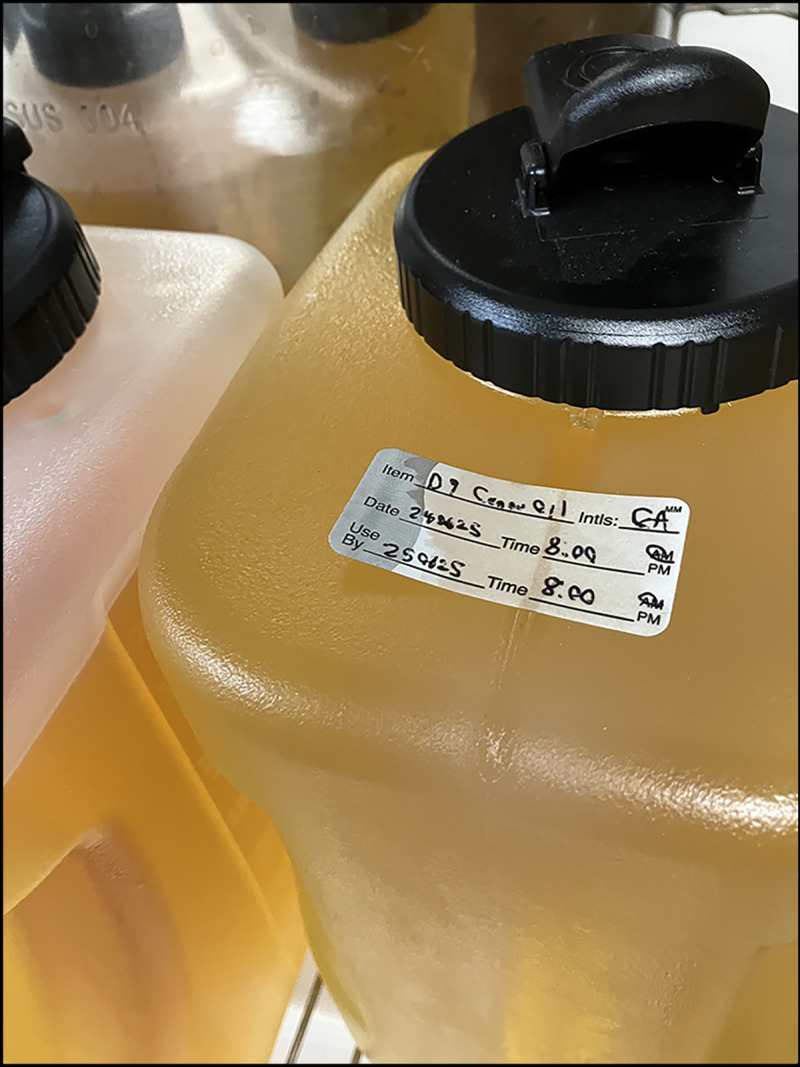
Containers* with hemp-derived Δ^9^-tetrahydrocannabinol–infused cooking oil used to prepare pizza and bread dough — Wisconsin, October 2024 Photo/Public Health Madison & Dane County **Abbreviation:** D9 = Δ^9^-tetrahydrocannabinol. * Containers are labeled as D9 oil, with the type of oil unclear.

On October 24, PHMDC contacted the restaurant owner. The owner was aware of the illnesses and agreed to close immediately. The same day, the restaurant owner called PHMDC and reported that a state-licensed business in a cooperative (i.e., shared) kitchen in the same building as the restaurant made edible products using hemp-derived Δ^9^-THC. The vendor infused cooking oil on-site with hemp-derived THC. Because of the possible THC involvement, PHMDC notified local police. During a third call with PHMDC, the restaurant owner reported that on October 22, the restaurant had run out of cooking oil and used oil from the cooperative kitchen to prepare dough that was served during October 22–24. The owner initially thought the oil was plain canola oil but later realized it might have been infused with THC. Oil from a large, labeled storage container (Figure 1), in the same area where the owner found the oil he used, tested positive for THC using the qualitative Duquenois-Levine reagent test. A police investigation concluded that the provision of THC-contaminated food to customers was unintentional, and no criminal charges were pursued. The restaurant reopened on October 26 after cleaning and sanitizing following standards in the Wisconsin Food Code.*

## Public Health Response

On October 24, PHMDC posted a food and symptom history questionnaire on its website. The link was distributed widely through a press release and on social media. All persons who experienced symptoms after eating food from the restaurant during October 22–24 were asked to complete the questionnaire. Respondents were asked to provide the names of other persons who had eaten food from the restaurant and become ill. The hospital provided PHMDC with the names of nine patients who sought care during October 23–24 after eating at the restaurant. On October 25, PHMDC contacted persons on this list who had not completed a questionnaire and asked them to complete the questionnaire.

**FIGURE 2:**
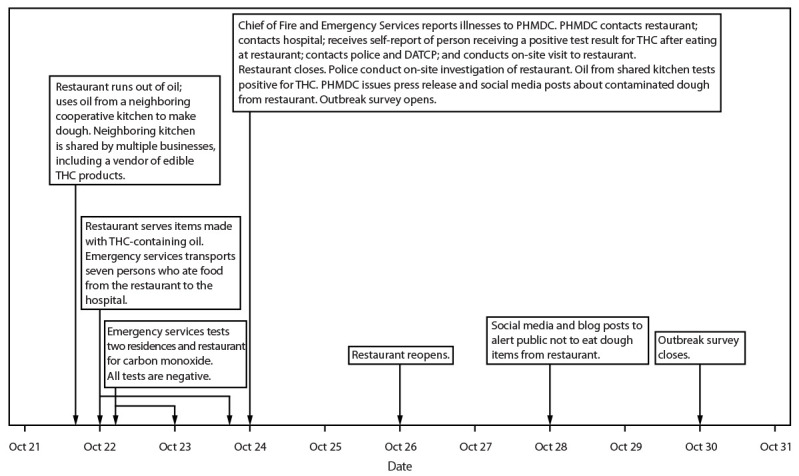
Timeline of events in an outbreak of tetrahydrocannabinol intoxication from food at a restaurant — Wisconsin, October 2024 **Abbreviations:** DATCP = Wisconsin Department of Agriculture, Trade and Consumer Protection; PHMDC = Public Health Madison & Dane County; THC = tetrahydrocannabinol.

During October 24–30, PHMDC received 208 responses; 101 were excluded from analysis because they contained 1) mostly incomplete information, 2) the name of a fictional character, 3) names or addresses that included marijuana or THC-related words, or 4) information that indicated the person did not actually eat food from the restaurant. The remaining 107 responses were considered valid (Figure 2). An outbreak-associated case of THC intoxication was defined as at least one of the following signs or symptoms (Table): dizziness, sleepiness, anxiety, short term memory impact or time distortion, increased heart rate, nausea, paranoia, panic attack, increased blood pressure, vomiting, or hallucinations in a person who ate pizza, garlic bread, cheese bread, or a grinder (submarine sandwich) purchased from the restaurant during October 22–24, with symptom onset within 5 hours of eating the restaurant’s food. Eighty-five persons met the case definition for THC intoxication. Median time from eating food to symptom onset was 1 hour (range = 0–4 hours). Forty-seven persons meeting the case definition were male, and 38 were female; median age was 43 years (range = 1–91 years). Thirty-three persons consulted a health care provider (28 in a hospital ED and five elsewhere), and three were hospitalized for at least 1 night. Based on self-reporting in the questionnaire comments and information from the hospital, 15 persons received a positive test result for THC (Supplementary Figure). Information about whether THC testing was performed was not available from the other 70 respondents. Eight persons (9%) were children and adolescents aged <18 years, none of whom was hospitalized. Dizziness was the only symptom reported for the youngest child (aged 1 year) with “not sure” indicated for all other symptoms in the questionnaire. Four children, aged <13 years (range = 3–8 years), had symptoms of dizziness (four), nausea (four), sleepiness (four), vomiting (three), anxiety (two), paranoia (one), short-term memory impact or time distortion (one), hallucinations (one), head or neck pain (one), and a heavy head (one). Respondents were asked to provide the names of other persons who ate food from the restaurant and became ill; eight additional persons who became ill after eating at the restaurant were identified, but none completed a questionnaire.

**TABLE:** Signs and symptoms reported by persons (N = 85) with outbreak-associated cases of tetrahydrocannabinol intoxication — Wisconsin, October 2024

Sign or symptom	Questionnaire response, no. (%)		
	Yes	No	Not sure
**Solicited in questionnaire**			
Dizziness	80 (94.1)	4 (4.7)	1 (1.2)
Sleepiness	76 (89.4)	6 (7.1)	3 (3.5)
Anxiety	67 (78.8)	11 (12.9)	7 (8.2)
Short-term memory impact or time distortion	59 (69.4)	13 (15.3)	13 (15.3)
Increased heart rate	54 (63.5)	7 (8.2)	24 (28.2)
Nausea	52 (61.2)	30 (35.3)	3 (3.5)
Paranoia	42 (49.4)	26 (30.6)	17 (20.0)
Panic attack	29 (34.1)	37 (43.5)	19 (22.4)
Increased blood pressure	28 (32.9)	4 (4.7)	53 (62. 4)
Hallucinations	22 (25.9)	53 (62.4)	10 (11.8)
Vomiting	10 (11.8)	72 (84.7)	3 (3.5)
**Reported as free-text response**			
Felt high, drugged, or drunk	13 (15.3)	—*	—
Ataxia^†^	11 (12.9)	—	—
Dry mouth	10 (11.8)	—	—
Head or neck pain	9 (10.6)	—	—
Perception distortion^§^	9 (10.6)	—	—
Numbness	8 (9.4)	—	—
Blurred vision	4 (4.7)	—	—
Food cravings	4 (4.7)	—	—
Affected work	3 (3.5)	—	—
Allergy-like symptoms	3 (3.5)	—	—
Body temperature irregularities	3 (3.5)	—	—
Confusion	3 (3.5)	—	—
Difficulty breathing	3 (3.5)	—	—
Fainting, light headedness, or weakness	3 (3.5)	—	—
Impaired driving	3 (3.5)	—	—
Muscle spasms	3 (3.5)	—	—
Behavioral abnormalities	2 (2.4)	—	—
Difficulty concentrating	2 (2.4)	—	—
Thirst	2 (2.4)	—	—
Diarrhea	1 (1.2)	—	—
Heavy head	1 (1.2)	—	—
Light sensitivity	1 (1.2)	—	—
Low potassium	1 (1.2)	—	—
Red eyes	1 (1.2)	—	—
Suicidal acts	1 (1.2)	—	—

* As free text entries, “no” and “not sure” were not questionnaire options.

^†^ Examples of terms used by respondents to describe ataxia included loss of balance; unable to stand, walk, or move; and feeling unsteady.

^§^ Examples of terms used by respondents to describe perception distortion included false sense of reality, psychosis, felt like being in a dream, heightened hearing, and having an out-of-body experience.

On October 24, PHMDC issued a press release alerting the public via social media not to eat leftover pizza purchased from the restaurant during October 22–24. This information was updated on October 28 to include leftover grinders, garlic bread, and cheese bread. The updated information was shared with the public via social media and PHMDC’s blog, which is posted to the PHMDC website and emailed to approximately 3,000 subscribers.

## Discussion

Eighty-five persons who responded to a health department questionnaire reported symptoms consistent with THC intoxication that occurred within 5 hours after eating food from a pizza restaurant, including 15 who received a positive THC test result. Respondents were asked to provide the names of others who ate food from the restaurant and became ill, which identified eight additional ill persons. No leftover foods were tested for THC, no quantitative testing was performed on the oil, and no analytical study (e.g., case-control study) was conducted.

Although FDA has not found that any use of a cannabinoid, including THC, in food would be safe and lawful under the Federal Food, Drug, and Cosmetic Act,^†^ these products can be found in adult cannabis and hemp marketplaces, some of which seek to follow state, territorial, or tribal laws regarding their use. Increased availability of food items containing THC increases the possibility that children (*1*,*2*) and adults (*3*) might unknowingly consume these items and experience symptoms that lead them to seek medical care. Co-location of food establishments with businesses that make THC-infused products increases the risk that a THC-containing ingredient might be added to food, either intentionally or unintentionally. Health care providers, public health professionals, and emergency responders should consider THC intoxication in persons with sudden onset of symptoms such as dizziness, sleepiness, anxiety, altered reality perception, increased heart rate, nausea, or other symptoms of THC ingestion. Regulations regarding practices such as standard, clear labeling (*4*) and locked storage for ingredients containing THC, might decrease the risk for unintentional THC exposure at licensed food businesses.

## Footnotes

* Wisconsin Food Code | Agriculture, Trade and Consumer Protection | ATCP 75 Appendix^† ^Food and Drug Administration | Warning Letter | Latro Inc. - 698196 - 03/25/2025; Food and Drug Administration | Warning Letter | Mary Jane's Bakery Co. LLC - 678010 - 07/15/2024; Food and Drug Administration | Warning Letter | Earthly Hemps - 674916 - 07/15/2024
